# *Lactobacillus johnsonii* alleviates colitis by TLR1/2-STAT3 mediated CD206^+^ macrophages^IL-10^ activation

**DOI:** 10.1080/19490976.2022.2145843

**Published:** 2022-11-18

**Authors:** Ding-Jia-Cheng Jia, Qi-Wen Wang, Ying-Ying Hu, Jia-Min He, Qi-Wei Ge, Ya-Dong Qi, Lu-Yi Chen, Ying Zhang, Li-Na Fan, Yi-Feng Lin, Yong Sun, Yao Jiang, Lan Wang, Yan-Fei Fang, Hui-Qin He, Xiong-E Pi, Wei Liu, Shu-Jie Chen, Liang-Jing Wang

**Affiliations:** a Department of Gastroenterology, Second Affiliated Hospital of Zhejiang University School of Medicine, Hangzhou, China; b Institution of Gastroenterology, Zhejiang University, Hangzhou, China; c Department of Gastroenterology, Sir Run Run Shaw Hospital, Zhejiang University, Hangzhou, China; d Department of General Practice, Sir Run Run Shaw Hospital, Zhejiang University, Hangzhou, 310058, China; e Institute of Plant Protection and Microbiology, Zhejiang Academy of Agriculture Sciences, Hangzhou, China; f Cancer Center, Zhejiang University, Hangzhou, China

**Keywords:** Lactobacillus johnsonii, Ulcerative colitis, Macrophages, STAT3, IL-10

## Abstract

Imbalance of gut microbiota homeostasis is related to the occurrence of ulcerative colitis (UC), and probiotics are thought to modulate immune microenvironment and repair barrier function. Here, in order to reveal the interaction between UC and gut microbiota, we screened a new probiotic strain by 16S rRNA sequencing from Dextran Sulfate Sodium (DSS)-induced colitis mice, and explored the mechanism and clinical relevance. *Lactobacillus johnsonii* (*L. johnsonii*), as a potential anti-inflammatory bacterium was decreased colonization in colitis mice. Gavage *L. johnsonii* could alleviate colitis by specifically increasing the proportion of intestinal macrophages and the secretion of Il-10 with macrophages depleted model and in *Il10^−/−^* mice. We identified this subset of immune cells activated *by L. johnsonii* as CD206^+^ macrophages^IL−10^. Mechanistically, *L. johnsonii* supplementation enhanced the mobilization of CD206^+^ macrophages^IL−10^ through the activation of STAT3 *in vivo* and *in vitro*. In addition, we revealed that TLR1/2 was essential for the activation of STAT3 and the recognition of *L. johnsonii* by macrophages. Clinically, there was positive correlation between the abundance of *L. johnsonii* and the expression level of *MRC1, IL10* and *TLR1/2* in UC tissues. *L. johnsonii* could activate native macrophages into CD206^+^ macrophages and release IL-10 through TLR1/2-STAT3 pathway to relieve experimental colitis. *L. johnsonii* may serve as an immunomodulator and anti-inflammatory therapeutic target for UC.

## Background

Ulcerative colitis (UC) is a subtype of inflammatory bowel disease (IBD), characterized by chronic nonspecific inflammation of the rectum and colon.^1,2^ Both adaptive and innate immunity are considered to be involved in this chronic inflammation process.^3^ Gut microbiota played vital roles in the occurrence and development of IBD.^4,5^ *Proteus mirabilis* as a key bacterium for Crohn’s disease could trigger inflammatory responses.^6^ Extracellular vesicles of *Fusobacterium nucleatum* compromise intestinal barrier in UC through targeting RIPK1-mediated cell death pathway.^7^ Gut microbiota had been discovered with the deepening of sequencing technology, while the mechanism remains to be elucidated. *Lactobacillus* and *Bifidobacterium* are two recognized genera of probiotics.^8,9^ We have previously reported the anti-inflammatory effect of *Bifidobacterium adolescentis* in UC.^10^ *Lactobacillus johnsonii* (*L. johnsonii*) is classified in the genus *Lactobacillus*. Study has shown that *L. johnsonii* as a potential beneficial bacteria could improve memory impairment through the brain-gut axis^11^ and regulate metabolic-related diseases.^12,13^

The regulatory role of the immune system is considered to be a bridge linking the gut microbiota and the host.^14,15^ A large number of immune cells such as macrophages, dendritic cells (DCs), T cells, B cells, and NK cells are colonized in the intestinal lamina propria. Macrophages, as a member of natural immunity, are widely involved in tissue repair, regeneration, and inflammation.^16^ In addition to the conventional use of M1-like and M2-like to differentiate macrophages, the updated recommendation is to use the marker or cytokine identifying the macrophages rather than a phenotypic designation.^17^ The mannose receptors (CD206^+^) macrophages, as surface markers of anti-inflammatory macrophages, have been reported to distinguish colonic macrophage populations between health and IBD.^18^

In this study, we uncovered that the abundance of *L. johnsonii* was lessened in colitis by 16S rRNA sequencing. Supplement with *L. johnsonii* alleviated DSS-induced colitis. *L. johnsonii* could stimulate the activation of CD206^+^ macrophages through the TLR1/2-STAT3 pathway and promote the secretion of the anti-inflammatory cytokine IL-10. The abundance of *L. johnsonii* in UC patients was positively correlated with the level of *IL10* and *TLR1/2*. Our findings provide insights for the gut microbiota to regulate the host immune system. *L. johnsonii*, as a probiotic, could be a potential strategy for UC treatment.

## Results

### *L.* *johnsonii* relieved

**Figure 1.**
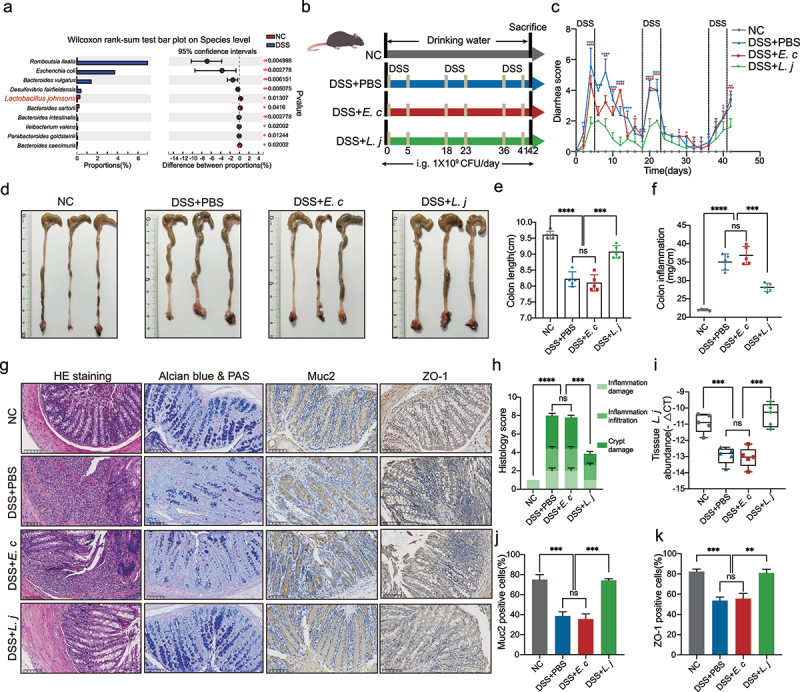
*L. johnsonii* relieved DSS-induced colitis and maintained intestinal mucosal barrier.

In order to explore the potential gut microbiota associated with UC, we constructed a DSS-induced chronic colitis model, and performed the fecal 16S rRNA sequencing. Fecal bacterial symbiotic richness and diversity (α-diversity) were significantly decreased, which was evident in the Sobs, Ace, and Chao index in colitis mice (**Figure S1A-C**). Furthermore, we performed principal component analysis (PCA) on the OTU level, which showed differences in microbial structure (β-diversity) between normal and inflammatory environments (**Figure S1D**). We then focused on the potential beneficial bacteria by the comparison with normal and inflammatory states, which indicated the abundance of *L. johnsonii* was depleted in colitis mice (Figure 1a**, Figure S1E**). The qPCR results confirmed the lower abundance of intestinal colonized *L. johnsonii* in both chronic (**Figure S1F**) and acute (**Figure S1G**) colitis.

**a**, Analysis of differences between NC and DSS groups on the species level from 16S rRNA sequencing. **b**, Schematic diagram showing that the process of DSS-induced chronic colitis. **c**, Diarrhea score was recorded according to rectal bleeding and diarrhea. **d**, Representative colon images of the groups with free drinking water (NC), gavaging PBS (DSS+PBS), gavaging *E. coli* MG1655 (DSS+*E. c*) and gavaging *L. johnsonii* (DSS+*L. j*). **e**, Colon length was analyzed in four groups. **f**, Colon inflammation was analyzed in four groups^10^. **g**, Representative pictures of HE staining, Alcian blue & PAS staining, the Muc2 and ZO-1 immunohistochemistry. Scale bar, 100 µm. **h**, Cumulative scores including inflammation damage, inflammation infiltration and crypt damage were measured. **i**, Bacteria genomic DNA was extracted from feces of mice with DSS-induced chronic colitis. The abundance of *L. johnsonii* was tested by RT-qPCR. **j-k**, The relative intensities of IHC (Figure 1g) were quantified by ImageJ software in 5 random fields in each group. Data are presented as mean ± SD, n = 5. **, P < .01; ***, P < .001; ****, P < .0001; ns no significant. Wilcoxon rank-sum test **(a)**, ANOVA test **(e-f, h-k)**.

To determine the role of *L. johnsonii* in the development of colitis, we constructed DSS-induced chronic colitis and gavaged with PBS (DSS+PBS), *E. coli* MG1655 (DSS+*E. c*) or *L. johnsonii* (DSS+*L. j*) (Figure 1b). Results demonstrated that *L. johnsonii* alleviated the severity of diarrhea in colitis mice as compared with PBS control or *E. coli* group (Figure 1c). In addition, *L. johnsonii* could reduce the degree of intestinal inflammation and subsequent intestinal shrinkage (Figure 1d-f). The weight of the spleen in the DSS+*L. j* group was also decreased **(Figure S1H-I**). Hematoxylin-Eosin (HE) staining showed lower cumulative scores characterized by loss of epithelium, infiltration of inflammatory cells, depletion of goblet cells and damage of crypt in *L. johnsonii* fed mice (Figure 1g-h). The mRNA expression levels of inflammatory factors including *Nos2, Tnfα, Il1β*, and *Il12a* were reduced in DSS+*L. j* group **(Figure S1J**).

To explore the effect of *L. johnsonii* inoculation on the intestinal microbiota homeostasis, we performed fecal 16S rRNA sequencing from DSS+PBS, DSS+*E. c* and DSS+*L. j* groups. PCA revealed that daily *L. johnsonii* supplementation induced significant changes in the composition of the gut microbiota **(Figure S1K**). The relative abundance of bacteria on the phylum level showed that *Bacteroidota* was enriched after *L. johnsonii* gavaged, whereas *Firmicutes* and *Actinobacteriota* were decreased **(Figure S1L, N**). The imbalance of *Bacteroidetes/Firmicutes* (B/F) ratio, which reflected intestinal homeostasis, was associated with diseases such as obesity and IBD.^19^ Gavage *L. johnsonii* increased the B/F ratio **(Figure S1M**). The relative abundance of bacteria on the family level showed that *L. johnsonii* increased the levels of *Prevotellaceae*^20^ and order *Clostridia*,^21^ which were closely related to the secretion of short chain fatty acids (SCFAs), and decreased the levels of *Lachnospiraceae, Oscillospiraceae* and *Erysipelotrichaceae* **(Figure S1O**). We also determined gavage *L. johnsonii* could colonize in the colon tissues by qPCR analysis (Figure 1i).

Mucin secreted by goblet cells is an important part of colonic mucosal barrier.^22^ Goblet cells were less damaged in the DSS+*L. j* group with staining of Alcian blue & PAS and Muc2 (Figure 1g, j).^23^ We also found that the intestinal mucosa barrier stained by ZO-1 was protected with *L. johnsonii* (Figure 1g, k). Collectively, the results indicated that *L. johnsonii* could relieve chronic colitis and maintain the colonic mucosal barrier.

### Macrophages immune response was involved in the anti-inflammatory effect by *L.* *johnsonii* in colitis

**Figure 2.**
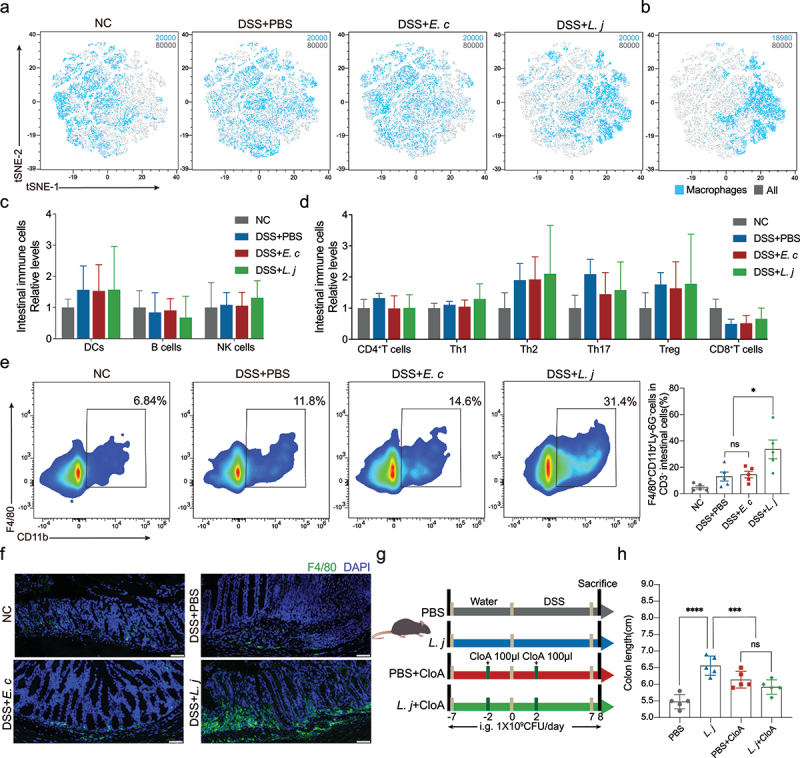
Macrophages immune response was involved in the anti-inflammatory effect by *L. johnsonii* in colitis.

When intestinal barrier was damaged by inflammation, the microbiota entered the lamina propria and interacted with the immune cells colonized.^24,25^ Therefore, we examined immune cells response in colon tissues by flow cytometry **(Figure S2A-B**), and the cell compositions by tSNE dimensionality reduction assay **(Figure S2C**). The cell compositions were separated into different cell clusters (Figure 2a). The number of macrophages was significantly increased in the DSS+*L. j* group **(Figure S2D)** by the gating strategy (Figure 2b). Flow cytometry and immunofluorescence analysis confirmed that *L. johnsonii* supplementation could increase the proportion of colonized macrophages in the intestine (Figure 2e-f). However, no significant changes had been observed in other types of immune cells including DCs, B cells, NK cells, CD4^+^ T cells, Th1, Th2, Th17, Treg or CD8^+^ T cells (Figure 2c-d). To further confirm the anti-inflammatory effect of *L. johnsonii* mediated by macrophages, we constructed a DSS-induced acute colitis model **(Figure S3A)**. Consistent results showed that *L. johnsonii* supplementation could reduce the degree of intestinal inflammation, cumulative scores by HE staining and the expression of inflammatory factors **(Figure S3B-F)**, while increased the number of macrophages colonized in the intestine **(Figure S3G)**.

**a**, tSNE dimensionality reduction analysis of CD45^+^ immune cells. 20000 cells were randomly selected from each group, a total of 80000 cells. **b**, tSNE dimensionality reduction analysis of macrophages cluster (18980 cells) in CD45^+^ immune cells (80000 cells). **c**, Frequencies of DCs, B cells, NK cells from colon lamina propria in four groups were tested by multicolor flow cytometry. **d**, Frequencies of CD4^+^T cells, Th1, Th2, Th17, Treg and CD8^+^T cells from colon lamina propria in four groups were tested by multicolor flow cytometry. **e**, Flow cytometry representation of macrophages from colon lamina propria in four groups. **f**, Representative images of immunofluorescence staining of macrophages (dual F4/80^+^) in chronic colitis mice. Scale bars, 50 µm. **g**, Schematic diagram showing that the process of macrophages clearance model. **h**, Colon length was analyzed in four groups. Data are presented as mean ± SD, n = 5. *, P < .05; ***, P < .001; ****, P < .0001; ns no significant. ANOVA test **(c-e, i)**.

In order to explore whether macrophages were contributed to the abrogation of intestinal inflammation, we used clodronate liposomes to deplete phagocytic gut macrophages as previously reported^26–28^ (Figure 2g). The depletion of macrophages **(Figure S4B-C)** could attenuate the anti-inflammatory and colon length prolongation effect by *L. johnsonii* (Figure 2h**, Figure S4A)**. Supplement with *L. johnsonii* could not protect the infiltration of inflammation and the damage of crypts when chemical clearance of macrophages **(Figure S4D-E)**. Collectively, macrophages were involved in the anti-inflammatory process by *L. johnsonii* in colitis.

### *L.* *johnsonii* promoted CD206^+^macrophages^IL-10^ activation *in* *vivo* and *in* *vitro*

**Figure 3.**
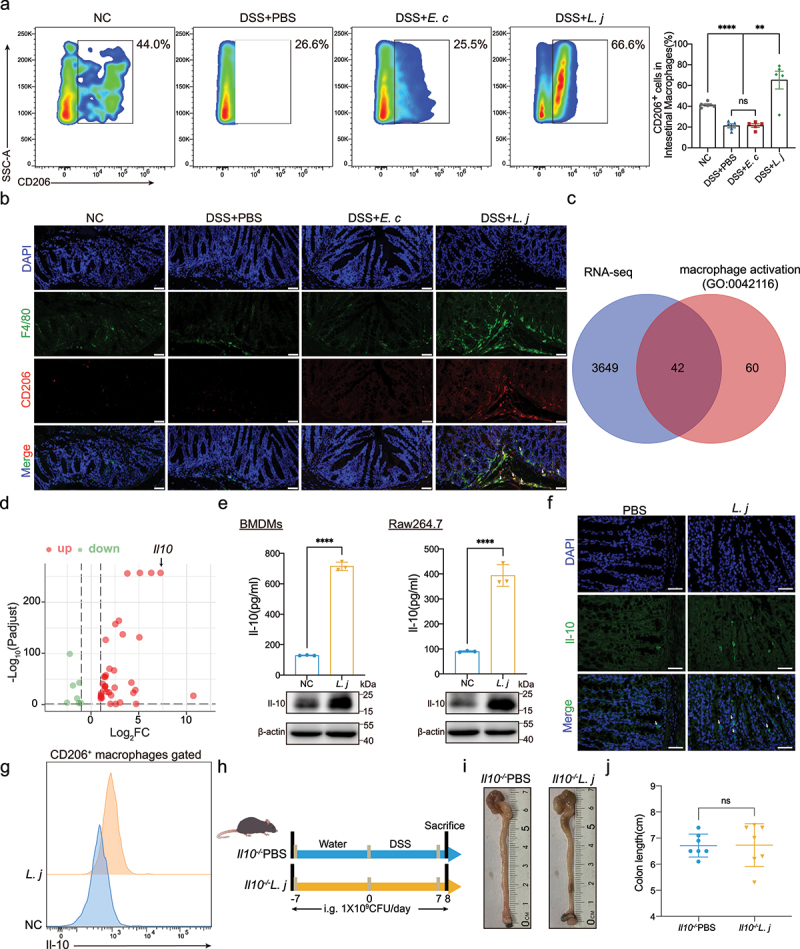
*L. johnsonii* promoted CD206^+^ macrophages^IL-10^ activation *in vivo* and *in vitro.*

Macrophages activation and polarization was involved in inflammation.^29^ Flow cytometry and immunofluorescence analysis showed that *L. johnsonii* could upregulate intestinal CD206^+^ macrophages in chronic colitis mice (Figure 3a-b), but had no obvious effect on CD11c^+^ macrophages **(Figure S5B)**. Similar result was observed in acute colitis **(Figure S5A, C)**. The activation of CD206^+^ macrophages in bone marrow-derived macrophages (BMDMs) and mouse macrophages cell line Raw264.7 were also observed after co-cultured with *L. johnsonii in vitro* **(Figure S5D-F)**. These results indicated that *L. johnsonii* could activate CD206^+^ macrophages *in vivo* and *in vitro*.

**a**, Flow cytometry representation of CD206^+^ macrophages from colon lamina propria in chronic colitis model. **b**, Representative images of immunofluorescence staining of CD206^+^ macrophages (dual F4/80^+^ CD206^+^) in chronic colitis mice. Scale bars, 50 µm. The white arrows indicated the positive stained cells. **c**, Venn diagram showed the intersecting of the RNA sequencing results and the macrophage activation gene set (GO:0042116). **d**, Volcano map of the differential gene expression patterns (Figure 3c) (n = 3 per group, fold change>2, logCPM>2, FDR<0.05). **e**, The protein expression of Il-10 of PBS control (NC) or *L. johnsonii* (*L. j*) treated BMDMs and Raw264.7 cells were examined by ELISA and western blot. **f**, Representative images of immunofluorescence staining of Il-10 in colon tissues of PBS control (PBS) or *L. johnsonii* (*L. j*) treated chronic colitis mice. Scale bars, 100 µm. The white arrows indicated the positive stained cells. **g**, Representative histograms of Il-10 expression by CD206^+^ macrophages were shown. **h**, Schematic diagram showing that the process of DSS-induced acute colitis in *Il10^−/−^* mice. **i**, Representative colon images of the groups with gavaging PBS (*Il10^−/−^*PBS), gavaging *L. johnsonii* (*Il10^−/−^L. j*). **j**, Colon length was analyzed in two groups. Data are presented as mean ± SD, n = 5–7. **, P < .01; ****, P < .0001; ns no significant. ANOVA test **(a)**, unpaired Student’s t test **(e, j).**

To explore the effector molecules underlying *L. johnsonii* relieving inflammation through CD206^+^ macrophages, we performed RNA sequencing in BMDMs after incubated with *L. johnsonii* or PBS for 24 hours. Results showed that 1846 genes in BMDMs were significantly upregulated, while 1845 genes were downregulated after incubated with *L. johnsonii* **(Figure S6A)**. Then we intersected this RNA sequencing results with the macrophage activation gene set (GO:0042116) (Figure 3c). In line of this, 37 genes were significantly up-regulated and 7 genes were down-regulated in BMDMs after treatment with *L. johnsonii* **(Figure S6B)**. The volcano map showed that *Il10* was the second up-regulated gene (Figure 3d). IL-10 played a regulatory role in IBD.^29^ *L. johnsonii* supplementation could increase the secretion of Il-10 in both BMDMs and Raw264.7 cells *in* *vitro* by ELISA and western blot assays (Figure 3e). Immunofluorescence detection showed that the content of Il-10 in colitis tissues was increased after *L. johnsonii* treatment (figure 3f). In particular, *L. johnsonii* could increase the expression of Il-10 in CD206^+^ macrophages in BMDMs (Figure 3g).

We then determined whether *L. johnsonii* exerts its anti-inflammatory role in *Il10* knockout mice. *L. johnsonii* could not alleviate colon shortening and inflammation in *Il10^−/−^* mice treated by DSS as expected (Figure 3h-j**, Figure S6C-D)**. We defined this macrophages subtype induced by *L. johnsonii* as CD206^+^ macrophages.^IL-10^ Taken together, *L. johnsonii* could activate intestinal CD206^+^ macrophages, thus promoting the secretion of IL-10 to relieve colitis.

### STAT3 signaling was essential for *L.* *johnsonii* activated CD206^+^macrophages^IL-10^

**Figure 4.**
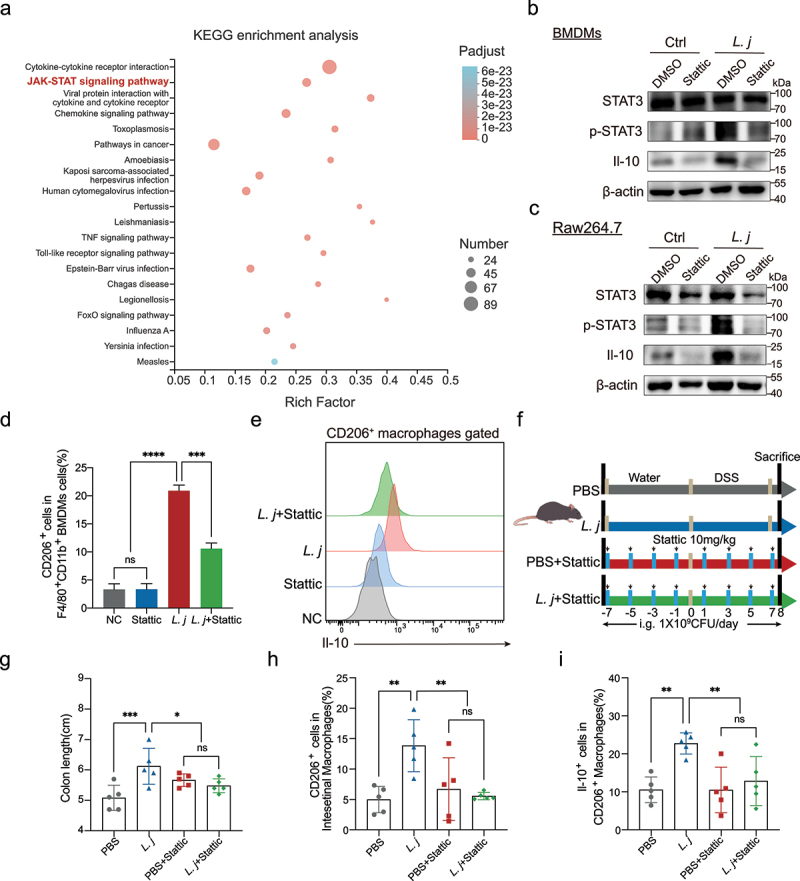
STAT3 signaling was essential for *L. johnsonii* activated CD206^+^macrophages^IL-10^.

To explore the underlying mechanism of *L. johnsonii* promoting the activation of CD206^+^ macrophages,^IL-10^ we performed KEGG enrichment analysis in the macrophages-related gene sets from the RNA sequencing **(Figure S6A)**, and found that the JAK-STAT signaling was significantly enriched (Figure 4a). Then we verified the target genes related to the JAK-STAT3 pathway in both BMDMs and Raw264.7 cells. The mRNA levels of *Pim1, Socs3, Cish*, and *Socs1* were significantly increased after *L. johnsonii* treatment **(Figure S7A-B)**. *L. johnsonii* could upregulate the phosphorylation level of STAT3 in BMDMs and Raw264.7 cells (Figure 4b-c). After treatment with Stattic, an STAT3 inhibitor,^30–32^ the expression of CD206 and Il-10 was downregulated (Figure 4d-e). We further constructed a STAT3 inhibition model with Stattic in acute colitis, and found that the inactivation of STAT3 abrogated the anti-inflammatory effect by *L. johnsonii* (figure 4f-g**, Figure S7C-E)**. There was no significant difference in the number of CD206^+^ macrophages^IL-10^ after Stattic was administered (Figure 4h-i**, Figure S7F-H)**. These results showed that STAT3 pathway was involved in the activation of CD206^+^ macrophages^IL-10^ by *L. johnsonii*.

**a**, KEGG enrichment analysis of macrophages related gene sets. The degree of color represented p adjust value and the size of node represented the gene number in this item. **b-c**, The protein expression of STAT3, p-STAT3, Il-10 was tested by western blot in both BMDMs and Raw264.7 cells. **d**, Percentage of CD206^+^ macrophages were analyzed by flow cytometry after BMDMs co-cultured with PBS control (NC), Stattic, *L. johnsonii* (*L. j)* or Stattic and *L. johnsonii* (*L. j+ *Stattic) (MOI = 100:1) for 24 hours. **e**, Representative histograms of Il-10 expression by CD206^+^ macrophages were shown after BMDMs co-cultured with PBS control (NC), Stattic, *L. johnsonii* (*L. j)* or Stattic and *L. johnsonii* (*L. j+ *Stattic) (MOI = 100:1) for 24 hours. **f**, Schematic diagram showing that the process of STAT3 inactivation model *in vivo*. **g**, Colon length was analyzed in four groups. **h**, Percentage of CD206^+^ macrophages were analyzed from colon lamina propria in STAT3 inactivation model. **i**, Percentage of Il-10^+^ cells were analyzed from CD206^+^ macrophages in STAT3 inactivation model. Data are presented as mean ± SD, n = 3–5. *, P < .05; **, P < .01; ***, P < .001; ****, P < .0001; ns no significant. ANOVA test **(d, g-i)**.

### TLR1/2 participated in the recognition of *L.* *johnsonii* by CD206^+^macrophages^IL-10^

**Figure 5.**
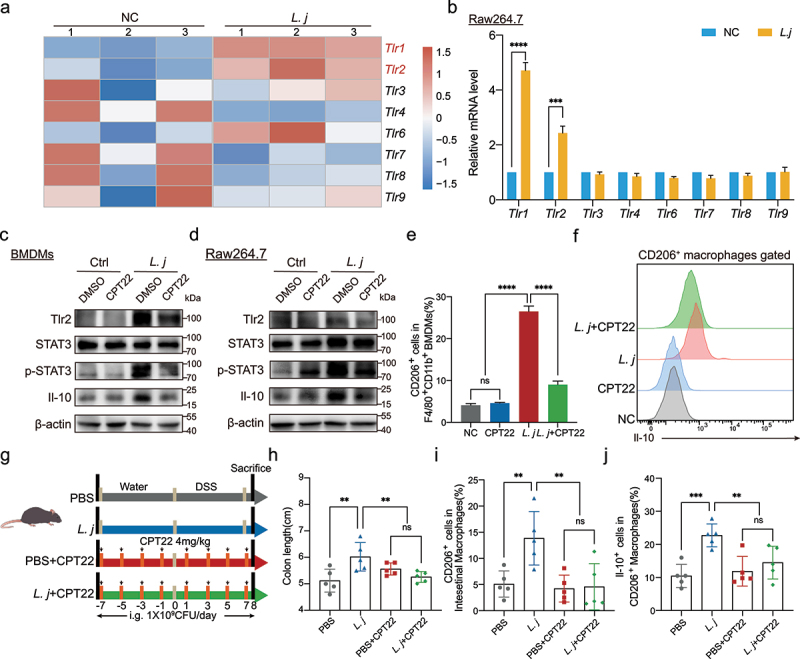
TLR1/2 participated in the recognition of *L. johnsonii* by CD206^+^macrophages^IL-10^.

Toll-like receptor family (TLRs) was closely involved in the interaction between microbiota and immune cells.^33,34^ In order to explore the pattern recognition receptors (PRRs) which macrophages recognized *L. johnsonii*, we filtered the relative expression of TLRs genes and found that *Tlr1* and *Tlr2* were significantly up-regulated in BMDMs and Raw264.7 cells after *L. johnsonii* stimulation (Figure 5a-b). There were no significant changes of *Tlr3, Tlr4, Tlr6, Tlr7, Tlr8* and *Tlr9* (Figure 5a-b).

**a**, Heat map representing the TLRs genes expression patterns between *L. johnsonii*-treated or PBS-treated BMDMs by Microarrays (n = 3 per group, fold change>2, logCPM>2, FDR<0.05). **b**, The mRNA levels of the TLRs *Tlr1, Tlr2, Tlr3, Tlr4, Tlr6, Tlr7, Tlr8* and *Tlr9* were evaluated in Raw264.7 cells after *L. johnsonii* treatment. **c-d**, The protein expression of Tlr2, STAT3, p-STAT3, Il-10 was tested by western blot in both BMDMs **(c)** and Raw264.7 cells **(d). e**, Percentage of CD206^+^ macrophages were analyzed by flow cytometry after BMDMs co-cultured with PBS control (NC), CPT22, *L. johnsonii* (*L. j)* or CPT22 and *L. johnsonii* (*L. j+ *CPT22) (MOI = 100:1) for 24 hours. **f**, Representative histograms of Il-10 expression by CD206^+^ macrophages were shown after BMDMs co-cultured with PBS control (NC), CPT22, *L. johnsonii* (*L. j)* or CPT22 and *L. johnsonii* (*L. j+ *CPT22) (MOI = 100:1) for 24 hours. **g**, Schematic diagram showing that the process of TLR1/2 inhibition model *in vivo*. **h**, Colon length was analyzed in four groups. **i**, Percentage of CD206^+^ macrophages were analyzed from colon lamina propria in TLR1/2 inhibition model. **j**, Percentage of Il-10^+^ cells were analyzed from CD206^+^ macrophages in TLR1/2 inhibition model. Data are presented as mean ± SD, n = 3–5. **, P < .01; ***, P < .001; ****, P < .0001; ns no significant. unpaired Student’s t test **(b)**, ANOVA test **(e, h-j)**.

It has been reported that TLR1 and TLR2, as a heterodimer, was involved in the identification of microorganisms.^35–37^ Therefore, we used CPT22, a TLR1/2 complex inhibitor,^38,39^ to evaluate its effect on the activation of macrophages. Results showed that the inhibition of TLR1/2 could block the activation of STAT3 and production of Il-10 in both BMDMs (Figure 5c) and Raw264.7 macrophages cells (Figure 5d) by *L. johnsonii*. We further explored the effect of TLR1/2 on CD206^+^ macrophages^IL-10^ activation by flow cytometry. Pretreatment with CPT22 significantly inhibited the expression of CD206 and the secretion of Il-10 (Figure 5e-f). The downstream targets of JAK-STAT3 pathway including *Pim1, Socs3, Cish* and *Socs1* were suppressed by pretreatment with TLR1/2 inhibitor **(Figure S8A-B)**. Similarly, blocking of TLR1/2 attenuated the anti-inflammatory effect by *L. johnsonii* in DSS-induced acute colitis (Figure 5g-h**, Figure S8C-E)**. There was no significant difference in the number of CD206^+^ macrophages^IL-10^ (Figure 5i-j**, Figure S8F-G)**. These results indicated that TLR1/2 participated in the recognition of *L. johnsonii* by macrophages, and which was required for *L. johnsonii* promoting the activation of CD206^+^ macrophages^IL-10^.

### The abundance of *L.* *johnsonii* was positively correlated with *MRC1, IL10* and *TLR1/2* in UC patients

**Figure 6.**
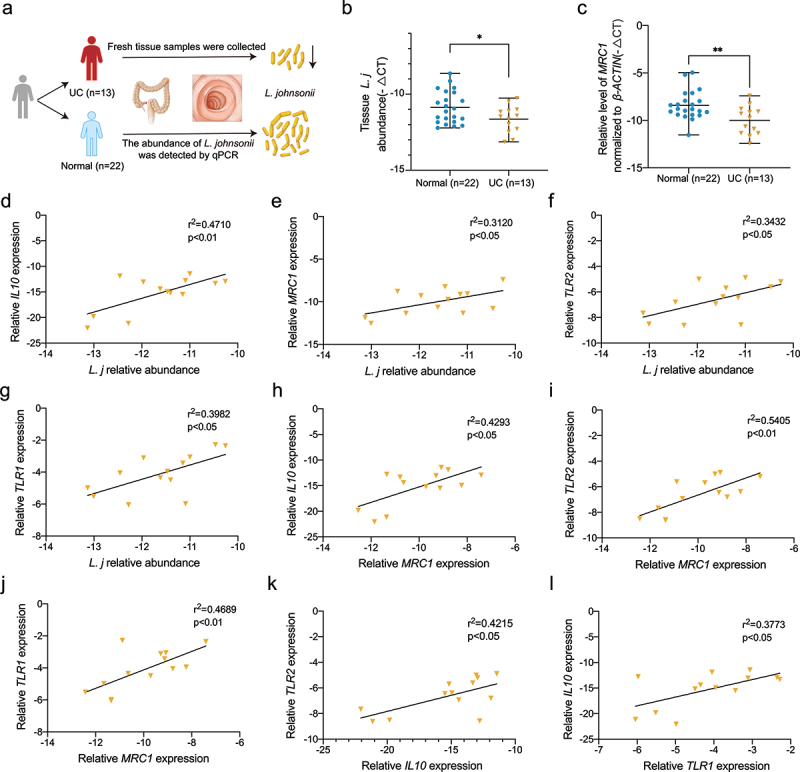
The abundance of *L. johnsonii* was positively correlated with *MRC1, IL10* and *TLR1/2* in UC patients.

To uncover the clinical relevance of *L. johnsonii* and UC, we assessed the abundance of *L. johnsonii* in UC tissues (n = 13) and normal tissues (n = 22) (Figure 6a) and found that the abundance of *L. johnsonii* was downregulated in UC patients (Figure 6b). Consistent observations were observed in colitis mice (Figure 1i**, Figure S1F-G)**. Furthermore, we found that the expression of human Mannose receptor C-type 1(*MRC1*), the homologous genes to CD206 in mouse, was decreased in UC patients (Figure 6c).

**a**, Schematic diagram showing that tissue samples from 13 patients with ulcerative colitis and 22 normal people were collected. **b**, Bacteria genomic DNA was extracted from colon tissues of UC patients and healthy people. The abundance of *L. johnsonii* was tested by RT-qPCR. **c**, The mRNA expression level of *MRC1* were evaluated in UC patients and healthy people. **d**, The correlation of *L. johnsonii* and *IL10* were analyzed. **e**, The correlation of *L. johnsonii* and *MRC1* were analyzed. **f**, The correlation of *L. johnsonii* and *TLR2* were analyzed. **g**, The correlation of *L. johnsonii* and *TLR1* were analyzed. **h**, The correlation of *MRC1* and *IL10* were analyzed. **i**, The correlation of *MRC1* and *TLR2* were analyzed. **j**, The correlation of *MRC1* and *TLR1* were analyzed. **k**, The correlation of *IL10* and *TLR2* were analyzed. **i**, The correlation of *TLR1* and *IL10* were analyzed. Data are presented as mean ± SD, n = 13–22. *, P < .05; **, P < .01. Mann Whitney test **(a-b)**, Spearman correlation analysis **(d-l)**.

Moreover, the abundance of *L. johnsonii* was positively correlated with the expression of *IL10, MRC1*, and *TLR1/2* (Figure 6d-g). There were positive relationships among the expression of *MRC1, IL10* and *TLR1/2* (Figure 6h-l). Summarily, our results indicated that *L. johnsonii* colonization was reduced in UC patients, and its abundance was positively correlated with *MRC1, IL10* and *TLR1/2*.

## Discussion

In this study, we reported that *L. johnsonii*, as a member of the *Lactobacillus*, was depleted in inflammatory intestine. *L. johnsonii* supplementation could alleviate DSS-induced acute and chronic colitis. This view was testified by Charlet *et al*., who found that mixed gavage of *L. johnsonii* and *B. thetaiotaomicron* alleviated DSS-induced acute colitis.^40^ Recent study reported that *L. johnsonii* reduced *Citrobacter rodentium*-induced colitis by regulating inflammation and endoplasmic reticulum stress.^41^ However, the interaction between *L. johnsonii* and the host immune system have not been clarified.

We found that the anti-inflammatory effects of *L. johnsonii* were mediated by macrophages. We then defined the *L. johnsonii* induced population of anti-inflammatory macrophages as CD206^+^ macrophages^IL-10^ based on the surface marker and secreted cytokine. As a traditional surface marker of M2-like macrophages, CD206 is participated in the anti-inflammatory process.^42^ We further demonstrated the importance of IL-10 in *L. johnsonii* induced inflammation suppression by RNA sequencing and *Il10* knock out mice. IL-10 could directly affect the mucus production and properties of goblet cells.^43^ *Il10^−/−^* mice were thought to be defective in colonic Muc2 synthesis.^44^ This is also consistent with our findings that *L. johnsonii* supplementation could restore barrier function by increasing the secretion of IL-10.

The activation effect of *L. johnsonii* on CD206^+^ macrophages^IL-10^ was mediated by STAT3 in our observations. The activation of STAT3 is crucial for mucosal repair in innate immunity^45^ and the activation of macrophages.^46^ The JAK-STAT3 pathway is often considered to be a pro-inflammatory signaling pathway in IBD,^47^ and inhibitors of JAK2 have been approved for clinical treatment of UC.^48^ JAK-STAT3 signaling may play diverse function depending on the microenvironment conditions. We found that the activation of STAT3 was involved in the anti-inflammatory effect by promoting the CD206^+^ macrophages^IL-10^ activation. TLRs were related with the recognition of gut microbiota by immune cells. *L. johnsonii* was reduced in mice with Toll-like receptor 7 agonist imiquimod treatment.^49^ *L. johnsonii* N6.2 stimulated the innate immune response through Toll-like receptor 9 in Caco-2 cells.^50^ These suggests the connection between *L. johnsonii* and TLRs. We found that the activation of TLR1/2 was essential for the recognition of *L. johnsonii* by macrophages. TLR2 has been widely reported as a common pattern recognition receptor, and it often forms heterodimers with TLR1 or TLR6 to complete the recognition process together.^35–37^ In addition, we found that *L. johnsonii* colonization on colonic tissues from clinical UC patients was reduced. The abundance of *L. johnsonii* was positively correlated with the expression of *TLR1/2*.

**Figure 7.**
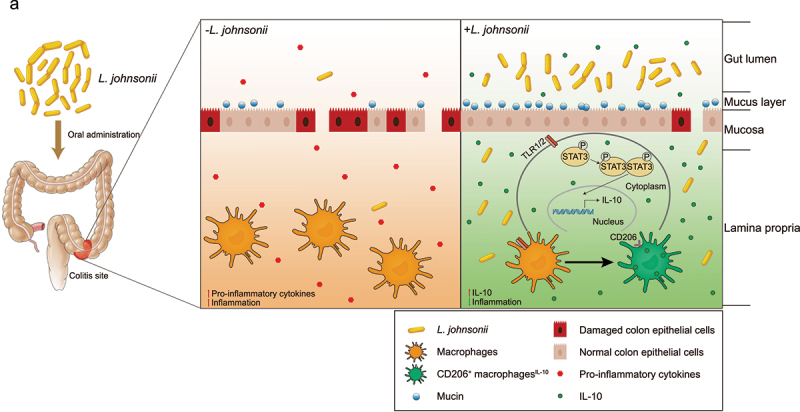
The schematic diagram of *L. johnsonii* relieving colitis.

In conclusion, we identified that *L. johnsonii* could promote the activation of CD206^+^ macrophages^IL-10^ through TLR1/2-STAT3 pathway, and relieved experimental colitis (Figure 7a).

**a**, Schematic diagram showing that the resident bacteria of *L. johnsonii* activates native macrophages into CD206^+^ macrophages and release IL-10 through TLR1/2-STAT3 pathway to relieve colitis.

## Methods

### Human sample collection

Tissue samples from 13 patients with ulcerative colitis and 22 normal people were obtained from Sir Run Run Shaw Hospital, Zhejiang University School of Medicine. Fresh tissue samples were collected during the health examination and immediately frozen in liquid nitrogen, stored in a − 80°C freezer. Ulcerative colitis patients were colonoscopically and pathologically diagnosed. The patients with ulcerative colitis were in active stage, without immunotherapy, and without antibiotics and probiotics in the past one month. Control individuals had no history of diarrhea or the use of antibiotics and probiotics in the past one month.

### DNA extraction and bacteria quantification

Bacterial DNA from mice feces was extracted using TIANGEN stool kits (DP328-02, TIANGEN, Beijing), and bacterial genomic DNA from human tissues or mice colon tissues were extracted using TIANGEN DNA kits (DP304-02, TIANGEN, Beijing). RT-qPCR was performed in ROCHE LightCycler®480 System (Rotor gene 6000 Software, Sydney, Australia). Each reaction was performed in triplicate with qPCR SYBR Green Master Mix (11198ES08, YEASON, China), primers and template DNA. Relative abundance was calculated using -ΔCt method. Universal Eubacteria 16S was used as an internal reference gene for stool samples.

*L. johnsonii: Forward: 5ʹ- TCGAGCGAGCTTGCCTAGATGA-3ʹ,*

*Reverse: 5ʹ- TCCGGACAACGCTTGCCACC-3ʹ;*

*Universal Eubacteria 16S: Forward: 5ʹ- CGGCAACGAGCGCAACCC-3ʹ,*

*Reverse: 5ʹ- CCATTGTAGCACGTGTGTAGCC-3.^51^*

### Bacteria strain and culture

*L. johnsonii* was independently isolated from pig feces by Zhejiang Academy of Agricultural Sciences and confirmed on the species level by 16S ribosomal RNA sequencing (V4 sequences). Bacterium were cultured in De Man, Rogosa and Sharpe (MRS) Medium (HB0384-5, hopebio, China) under an atmosphere of 10% H_2_, 10% CO_2_ and 80% N_2_ (AW500SG anaerobic workstations; ELECTROTEK, England) for 24 hours at 37°C. The nonpathogenic commensal intestinal bacteria, *E. coli* strain MG1655 (Biobw, China) was used as a negative control^52^ and was cultured in Luria-Bertani (LB) Medium (A507002 Sangon Biotech, China) at 37°C. The cultures were resuspended at a final concentration of 1 × 10^9^ CFU/ 200 µl.

### Animal models

Male C57BL/6 mice (6–8 weeks of age) were purchased from Shanghai SLAC Laboratory Animal, China. Male *Il10*^−/−^ mice (6–8 weeks of age) were provided by GemPharmatech Co., Ltd. All mice were housed in Sir Run Run Shaw Hospital, Zhejiang University School of Medicine under specific pathogen-free (SPF) conditions. Barrier environment maintains a 12-hour circadian rhythm, constant temperature and humidity, sufficient water and food. Before bacterial gavage, 2 mg/mL streptomycin was added to drinking water for 3 days to ensure the consistency of routine microbiota, as reported in a previous study.^53^

For chronic colitis model, Mice were randomized. Each group of mice was given 2% DSS (160110, MP Biomedicals) for 5 days and then drank normal water for 14 days, this process was repeated for three cycles. The mice in the NC group drank normal water freely throughout the modeling process. Mice in DSS+*L. j* and DSS+*E. c* groups were orally gavaged with 200 µl of *L. johnsonii* or *E. coli* MG1655 containing 1 × 10^9^ CFU per day, while mice in DSS+PBS group were given the same volume of sterile PBS.

For acute colitis model, Mice were randomized. Each group of mice was given 3% DSS (160110, MP Biomedicals) for 7 days and oral gavage lasted 14 days.

For clodronate liposomes-mediated macrophage deletion model,^26^ based on the acute colitis model, each mouse was injected intraperitoneally with 1 mg of clophosome-A (CloA), an anionic liposomal clodronate (F70101C-A, FormuMax, USA), in a volume of 100 µl 2 days prior to (day −2) and 2 days after (day 2) DSS exposure.^28^ Flow cytometry was used to detect the removal of macrophages residing in the intestine.

For STAT3 inactivation model *in vivo*, based on the acute colitis model, each mouse was injected intraperitoneally with 4 mg/kg Stattic (S7024, Selleckchem, Houston, TX, USA) once every two days.^54,55^

For TLR1/2 inhibition model *in vivo*, based on the acute colitis model, each mouse was injected intraperitoneally with 3 mg/kg CPT22 (S8677, Selleckchem, Houston, TX, USA) once every two days.^56,57^

Colorectal and spleen tissues were photographed and measured in length or weigh at the end of the experiment. Diarrhea scores were recorded daily, scoring bloody stools and diarrhea. Bloody stools: 0 = no bleeding, 2 = light bleeding, 4 = heavy bleeding; Diarrhea: 0 = well-shaped stools, 2 = soft and mushy stools, 4 = watery stools. The assessment of colonic inflammation was performed by normalization of colon weight (mg) by its length (cm).^10^

### Histopathological analysis

Colon tissues were formalin fixed overnight at room temperature and then embedded in paraffin. 5 µm sections were stained with hematoxylin and eosin (HE) for pathological analysis. The pathological score was assessed by the severity of inflammation, the degree of inflammatory involvement, and the degree of epithelial/crypt damage as previously reported.^58^ Calculate each parameter and sum to get the total score.

For immunohistochemistry, paraffin-embedded tissue sections were stained with anti-MUC2 antibody (GB14110, 1:1000, Servicebio, China), anti-ZO-1 antibody (GB111402, 1:1000, Servicebio, China) and visualized by DAB staining according to manufacturer’s instructions.

For immunofluorescence, paraffin-embedded tissues were stained with anti-F4/80 antibody (GB11027, 1:1000, Servicebio, China), anti-Il-10 antibody (GB11108, 1:1000, Servicebio, China) and anti-CD206 antibody (GB113497, 1:500, Servicebio, China). The slides were independently assessed by two researchers who were unaware of the nature of the samples.

### Isolation of colon lamina propria cells

As previously reported,^59^ residual lymphoid and adipose tissues were removed from colorectal tissue. Colon tissue was then cut into small pieces and placed in D-Hank’s buffer (MA0039, Meilunbio, China) containing 1 mM DTT (MB3047-1, Meilunbio, China) and 5 mM EDTA (MB2514, Meilunbio, China) at 37°C shaker 150 g for 30 minutes. Mucus-depleted colon tissues were cut into 1 mm pieces and further digested in Hank’s buffer (MA0041, Meilunbio, China) supplemented with 1 mg/ml type IV collagenase (A005318, Sangon, China) for 30 minutes at 37°C 150 g shaker. After complete digestion, the cell suspension was passed through a 200-mesh filter and then centrifuged at 700 g for 5 minutes. Isolated colonic lamina propria cells were collected for further flow cytometry analysis.

### Flow cytometry analysis

After the colonic lamina propria cells were counted, Fc receptors were blocked, 100 μl of the cell suspension was added with 1 μg of purified CD16/32 antibody (101320, Biolegend) and incubated on ice for 10 minutes. Then, cells were stained for live cells using Fixable viability Stain 510 (564406, BD Biosciences) for 30 minutes.

After termination, epifluorescence staining was performed using antibodies as described below: Alexa Fluor 700-CD45 (560510, BD Biosciences); Brilliant Violet 605-CD11b (101257, Biolegend); Brilliant Violet 421-F4/80 (565411, BD Biosciences); PE-I-A/I-E (557000, BD Biosciences); APC-CD206 (141708, Biolegend); PE-CY7-CD11c (117318, Biolegend); BUV395-Ly-6G (563978, BD Biosciences); Brilliant Violet 711-NK1.1 (108745, Biolegend); Brilliant Violet 785-CD45R/B220 (103246, Biolegend); PECP-CY5.5-CD3 (551163, BD Biosciences); Brilliant Violet 605-CD4 (100548, Biolegend); APC-CY7-CD8 (561967, BD Biosciences). Incubated in a refrigerator at 4°C for 30 minutes in the dark.

Then, cells were fixed in the dark at room temperature for 30 minutes (422101, Biolegend), centrifuged, and then the membranes were ruptured (421002, Biolegend). Intracellular staining was performed using antibodies as described below: PE-CY7-Foxp3 (560408, BD Biosciences); Brilliant Violet 421-Gata3 (563349, BD Biosciences); Alexa Fluor 647-T-bet (561267, BD Biosciences); PE-RORγt (IC6006P-025, R&D). The cells were incubated in the dark at room temperature for 60 minutes.

For the detection of Il-10 cytokines, after resuspending the cells with culture medium, added 2 μl PMA/Ionomycin, Activation Cocktails (423303, Biolegend) to 1 ml of cell suspension, incubated for 6 hours in a 37°C cell incubator, and then performed the above flow staining.

Samples were analyzed using Flow Cytometer (BD Biosciences). Subsequent analysis was performed with FlowJo software (Tree Star Inc., San Carlos, CA). tSNEs were calculated in FlowJo using the default settings for opt-SNE. For each set of analyses, individual cell subsets (CD45^+^ immune cells) from each group (NC, DSS+PBS, DSS+*E. c*, DSS+*L. j*) were randomly down-sampled to an equal number of events (20000 events).

### Bone marrow derived macrophages (BMDMs) and cell culture

After 8-week-old mice were sacrificed by cervical dislocation, they were disinfected by soaking in 75% alcohol for 15 minutes. The marrow cavities of the tibia and femur were opened and rinsed repeatedly with RPMI-1640 medium (Genom, China) supplemented with 10% FBS (Gibco) and 1% penicillin/streptomycin. The collected cells were cultured in RPMI-1640 medium containing mouse macrophage colony-stimulating factor (M-CSF, 20 ng/ml, Novoprotein, China), and the medium was renewed every 2 days. One week later, BMDMs were confirmed by flow cytometry for the expression of F4/80 (565411, BD Biosciences). The Raw264.7 mouse macrophage cell line was purchased from the American Type Culture Collection (ATCC, Manassas, VA, USA). Raw264.7 cells were cultured in RPMI-1640 medium (Genom, China) supplemented with 10% FBS (Gibco) and 1% penicillin/streptomycin. All cell lines were maintained at 37°C in a humidified 5% CO_2_ atmosphere.

BMDMs or Raw264.7 cells were seeded overnight in 6 well plates at 1 × 10^6^ cells per well and then cultured in antibiotic-free medium. BMDMs and Raw264.7 cells were incubated with *L. johnsonii* or *E. coli* at an MOI of 100:1 for 24 hours. For STAT3 inhibition experiments, Stattic (10 μM) was administered per six-well plate and incubated for 24 hours. For TLR1/2 inhibition experiments, CPT22 (10 μM) was administered per 6 well plate and incubated for 24 hours. Finally, BMDMs and Raw264.7 cells were digested for further flow cytometry analysis.

### RNA sequencing

After 24 hours of incubation with *L. johnsonii* (MOI = 100:1) or PBS, RNA from BMDMs were extracted and reverse transcribed into cDNA. RNA sequencing was paired-end at Shanghai MajorBio (China). Expression levels across the sample were expressed in RPKM (reads per kilobase per million). All differentially expressed genes were plotted with the “pheatmap” and “ggplot2” R packages. For KEGG enrichment analysis, p-value <0.05 was used as a threshold to determine significant enrichment for gene sets with the “clusterProfiler” R package.

### 16S rRNA sequencing

The fresh feces were collected for 16S rRNA sequencing on the 42nd day (before the mice were sacrificed). Bacterial genomic DNA was extracted from mouse feces as described above. Measured DNA concentration and checked DNA quality by 1% agarose gel electrophoresis. The V3-V4 hypervariable regions of the 16S ribosomal RNA gene was amplified by PCR system (GeneAmp 9700, ABI, USA) using primers 338 F and 806 R. PCR amplicons were purified by the AxyPrep DNA Gel Extraction Kit (Axygen Biosciences, Union City, CA, USA) and quantified by QuantiFluor™-ST (Promega, USA). Purified amplicons were sequenced on the Illumina MiSeq platform (Illumina, San Diego, USA) according to the standard protocols of Shanghai MajorBio (China). The raw data were decomposed and quality filtered by Trimmomatic and merged by FLASH. Operational taxonomic units (OTUs) were clustered using UPARSE (http://drive5.com/uparse/). We used the Ace, Chao, and Sobs diversity index to measure the species richness (α-diversity). The β-diversity was calculated using UniFrac distances and visualized using principal component analysis (PCA).

### RNA extraction and quantitative real-time PCR

RNA from mouse colon tissues or cells was extracted using Trizol reagent (Takara, Japan). PrimeScript™ RT reagent Kit (Takara, Japan) was used for reverse transcription. RT-qPCR was performed in Light Cycler®480 Real-Time PCR System (Roche) using SYBR Premix Ex Taq (Takara, Japan). cDNA was amplified by PCR under the following conditions: 95°C for 2 min, followed by 50 cycles of 95°C for 15s and 60°C for 30s. The mRNA expression of genes was analyzed using the specific primers listed in **(Table S1)**. The relative mRNA expression was calculated using -ΔCt method or the comparative cycle method (2^−ΔΔCt^). β-actin was used as internal control.

### Western blot analysis

Colon tissues or cells were lysed using RIPA extraction buffer (R0010, Solarbio, China). Protein concentrations were quantified using BCA protein assay kit (PC0020, Solarbio, China). Proteins were separated on 10% SDS polyacrylamide gels and then transferred to PVDF membranes. Membranes were blocked with quickblock solution (P0231, Beyotime, China) for 15 minutes, and then blocked with antibodies against STAT3 (4904, CST), p-STAT3 (9145, CST), Il-10 (A12255, ABclonal) and Tlr2 (13744, CST) at 4°C overnight. The membranes were then incubated with secondary antibody-conjugated HRP (1:10000, Abcam) for 1 hour at room temperature, and bands were visualized using an ECL kit (FD8000, Fdbio science, China). β-actin served as a loading control.

### Statistical analysis

All statistical analyses were performed using GraphPad Prism 9.0 software (GraphPad Software, Inc., La Jolla, CA, USA). The experiments were set up to use 3–7 samples/repeats for each experiment/group. Representative images for immunofluorescence staining, immunohistochemistry and immunoblot were shown. Each of these experiments was independently repeated over three times. Data was expressed as mean ± standard deviation (SD) or standard error of mean (SEM). Differences between groups were analyzed by unpaired Student’s t-test, one-way ANOVA test, Kruskal-Wallis test, Mann Whitney test or Wilcoxon matched-pairs signed-rank test. Spearman correlation analysis was used for correlation analysis. P value < .05 was considered statistically significant.

## Supplementary Material

Supplemental Material

## Data Availability

The RNA-sequencing dataset has been deposited in the Sequence Read Archive (SRA) accession number: PRJNA780203. Source data and reagents are available from the corresponding author on reasonable request.

## Authors’ information

These authors contributed equally: DJC Jia, QW Wang, YY Hu and JM He.

Corresponding Authors: Correspondence to LJ Wang, SJ Chen, W Liu.
