# Supplementary material for: Lactobacillus johnsonii alleviates colitis by TLR1/2-STAT3 mediated CD206+ macrophagesIL-10 activation
Source: Gut Microbes. 2022 Nov 18;14(1):2145843. doi: 10.1080/19490976.2022.2145843 (PMC9677986; doi:10.1080/19490976.2022.2145843)

Full Unedited Gel

Figure 3E

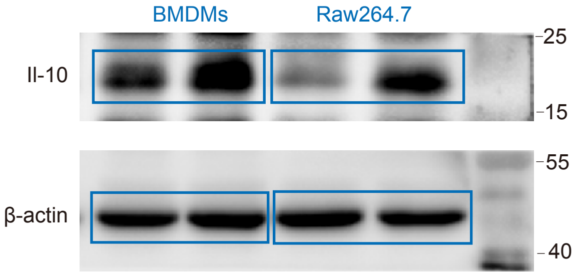

Figure 4B

The sample was divided equally, and two parts were and loaded separately on two gels.

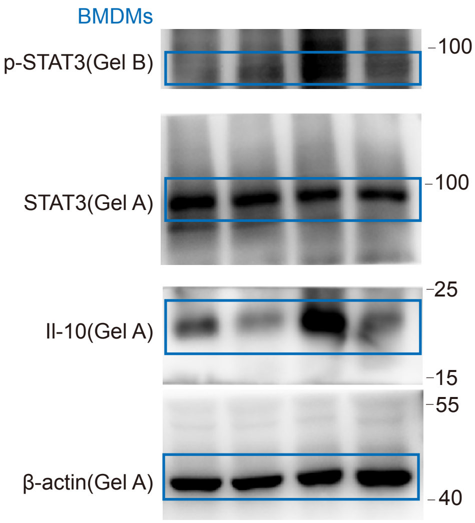

Figure 4C

The sample was divided equally, and two parts were and loaded separately on two gels.

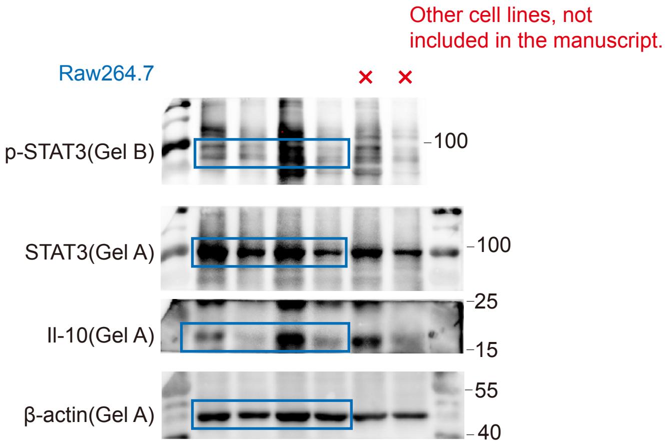

Figure 5C

The sample was divided equally, and three parts were and loaded separately on three gels.

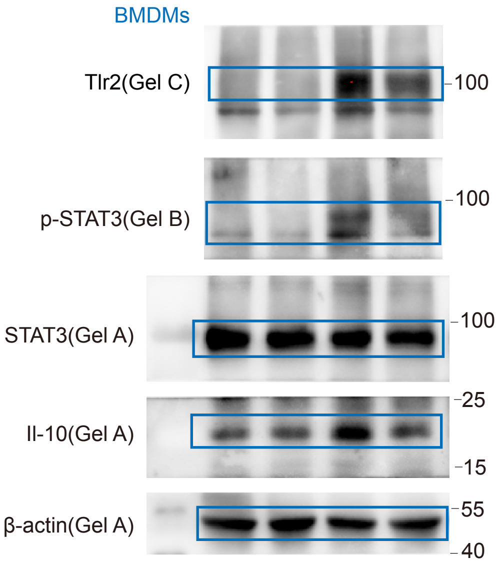

Figure 5D

The sample was divided equally, and three parts were and loaded separately on three gels.

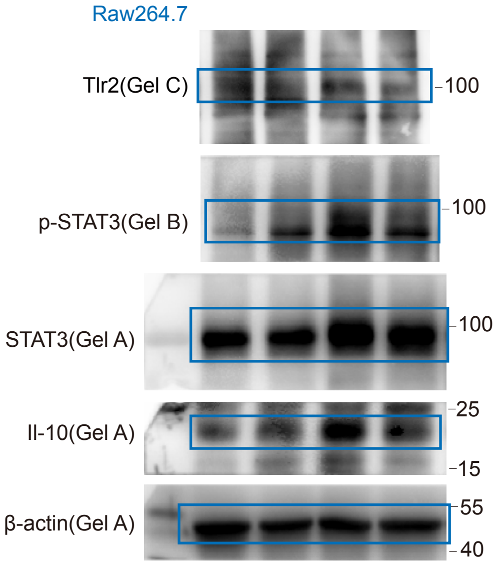

Supplement: Supplemental Material [file KGMI_A_2145843_SM4489.zip › Full unedited gel.pdf]
