# Supplementary material for: Lactobacillus johnsonii alleviates colitis by TLR1/2-STAT3 mediated CD206+ macrophagesIL-10 activation
Source: Gut Microbes. 2022 Nov 18;14(1):2145843. doi: 10.1080/19490976.2022.2145843 (PMC9677986; doi:10.1080/19490976.2022.2145843)
Supplement: Supplemental Material [file KGMI_A_2145843_SM4489.zip › Table S1.docx]

Primer list

Mouse *Nos2*: Forward: 5ʹ- ACATCGACCCGTCCACAGTAT -3ʹ,

Reverse: 5ʹ- CAGAGGGGTAGGCTTGTCTC -3ʹ;

Mouse *Tnfα*: Forward: 5ʹ- CCTGTAGCCCACGTCGTAG -3ʹ,

Reverse: 5ʹ- GGGAGTAGACAAGGTACAACCC -3ʹ;

Mouse *Il1β*: Forward: 5ʹ- GCAACTGTTCCTGAACTCAACT -3ʹ,

Reverse: 5ʹ- ATCTTTTGGGGTCCGTCAACT -3ʹ;

Mouse *Il12a*: Forward: 5ʹ- TGGTTTGCCATCGTTTTGCTG -3ʹ,

Reverse: 5ʹ- ACAGGTGAGGTTCACTGTTTCT -3ʹ;

Human *IL10*: Forward: 5ʹ- TCAAGGCGCATGTGAACTCC-3ʹ,

Reverse: 5ʹ- GATGTCAAACTCACTCATGGCT-3ʹ;

Mouse *Il10*: Forward: 5ʹ- CTTACTGACTGGCATGAGGATCA-3ʹ,

Reverse: 5ʹ- GCAGCTCTAGGAGCATGTGG-3ʹ;

Human *MRC1*: Forward: 5ʹ- GGGTTGCTATCACTCTCTATGC-3ʹ,

Reverse: 5ʹ- GCCTGATGCCAGGTTAAAGCA-3ʹ;

Mouse *CD206*: Forward: 5ʹ- CTCTGTTCAGCTATTGGACGC-3ʹ,

Reverse: 5ʹ- CGGAATTTCTGGGATTCAGCTTC-3ʹ;

Mouse *Pim1*: Forward: 5ʹ- CTGGAGTCGCAGTACCAGG-3ʹ,

Reverse: 5ʹ- CAGTTCTCCCCAATCGGAAATC-3ʹ;

Mouse *Socs3*: Forward: 5ʹ- ATGGTCACCCACAGCAAGTTT-3ʹ,

Reverse: 5ʹ- TCCAGTAGAATCCGCTCTCCT-3ʹ;

Mouse *Cish*: Forward: 5ʹ- ATGGTCCTTTGCGTACAGGG-3ʹ,

Reverse: 5ʹ- GGAATGCCCCAGTGGGTAAG-3ʹ;

Mouse *Socs1*: Forward: 5ʹ- CTGCGGCTTCTATTGGGGAC-3ʹ,

Reverse: 5ʹ- AAAAGGCAGTCGAAGGTCTCG-3ʹ;

Human *TLR1*: Forward: 5ʹ- TGAACCTCAAGCACTTGGACC-3ʹ,

Reverse: 5ʹ- CCCATAAGTCTCTCCTAAGACCA-3ʹ;

Mouse *Tlr1*: Forward: 5ʹ- TGAGGGTCCTGATAATGTCCTAC-3ʹ,

Reverse: 5ʹ- AGAGGTCCAAATGCTTGAGGC-3ʹ;

Human *TLR2*: Forward: 5ʹ-TTATCCAGCACACGAATACACAG-3ʹ,

Reverse: 5ʹ- AGGCATCTGGTAGAGTCATCAA-3ʹ;

Mouse *Tlr2*: Forward: 5ʹ-GCAAACGCTGTTCTGCTCAG-3ʹ,

Reverse: 5ʹ- AGGCGTCTCCCTCTATTGTATT-3ʹ;

Mouse *Tlr3*: Forward: 5ʹ-GTGAGATACAACGTAGCTGACTG-3ʹ,

Reverse: 5ʹ- TCCTGCATCCAAGATAGCAAGT-3ʹ;

Mouse *Tlr4*: Forward: 5ʹ-ATGGCATGGCTTACACCACC-3ʹ,

Reverse: 5ʹ- GAGGCCAATTTTGTCTCCACA-3ʹ;

Mouse *Tlr6*: Forward: 5ʹ-TGAGCCAAGACAGAAAACCCA-3ʹ,

Reverse: 5ʹ- GGGACATGAGTAAGGTTCCTGTT-3ʹ;

Mouse *Tlr7*: Forward: 5ʹ-ATGTGGACACGGAAGAGACAA-3ʹ,

Reverse: 5ʹ- GGTAAGGGTAAGATTGGTGGTG-3ʹ;

Mouse *Tlr8*: Forward: 5ʹ-GAAAACATGCCCCCTCAGTCA-3ʹ,

Reverse: 5ʹ- CGTCACAAGGATAGCTTCTGGAA-3ʹ;

Mouse *Tlr9*: Forward: 5ʹ-ATGGTTCTCCGTCGAAGGACT-3ʹ,

Reverse: 5ʹ-GAGGCTTCAGCTCACAGGG-3ʹ;

Mouse *β-actin*: Forward: 5ʹ- GAGACCTTCAACACCCCAGC-3ʹ,

Reverse: 5ʹ- GGAGAGCATAGCCCTCGTAGAT-3ʹ;

Human *β-ACTIN*: Forward: 5ʹ- AGAGCTACGAGCTGCCTGAC-3ʹ,

Reverse: 5ʹ- AGCACTGTGTTGGCGTACAG-3ʹ
