# Supplementary figures and images for: Lactobacillus johnsonii alleviates colitis by TLR1/2-STAT3 mediated CD206+ macrophagesIL-10 activation
Source: Gut Microbes. 2022 Nov 18;14(1):2145843. doi: 10.1080/19490976.2022.2145843 (PMC9677986; doi:10.1080/19490976.2022.2145843)

# Figure S1

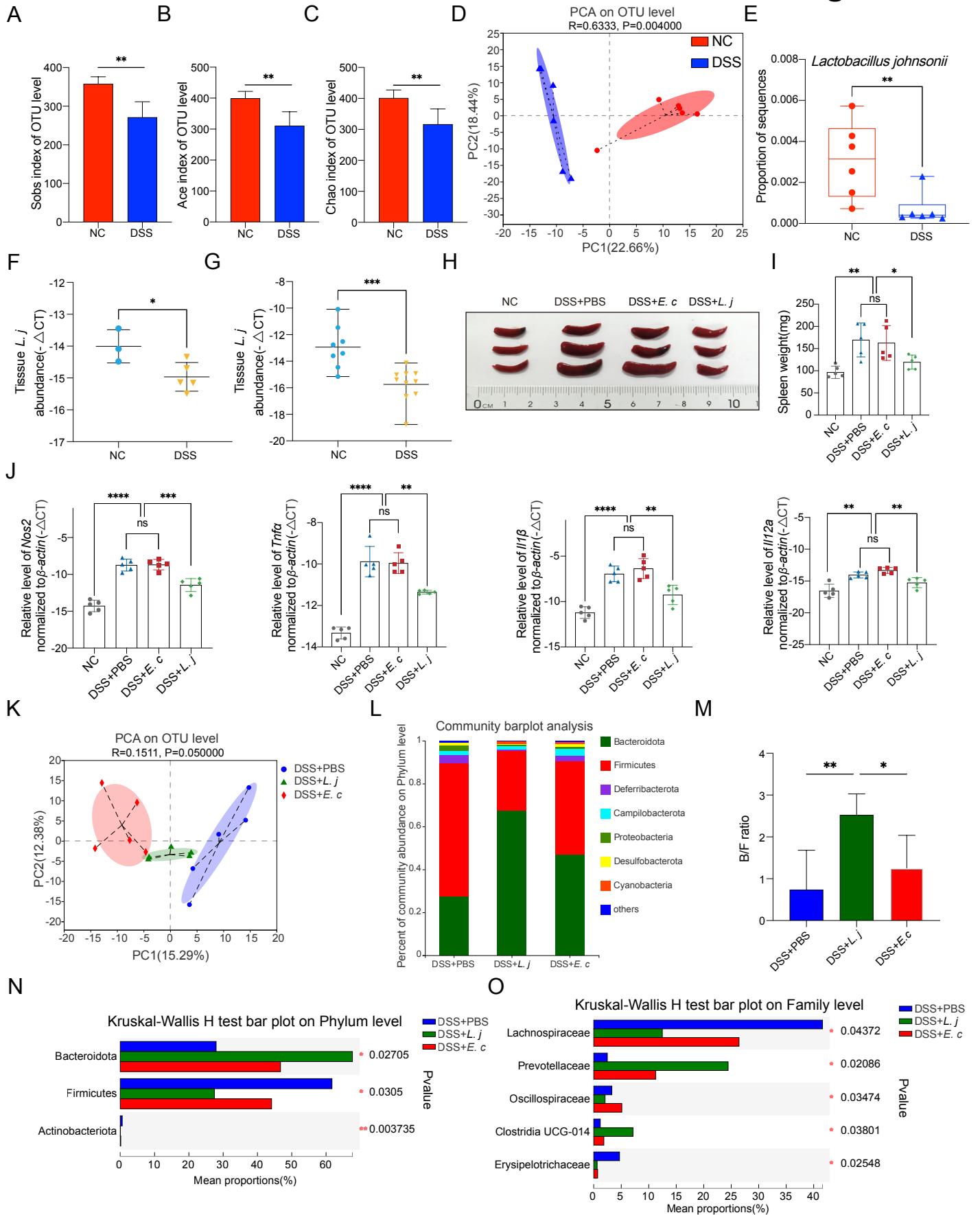

# Figure S2

A

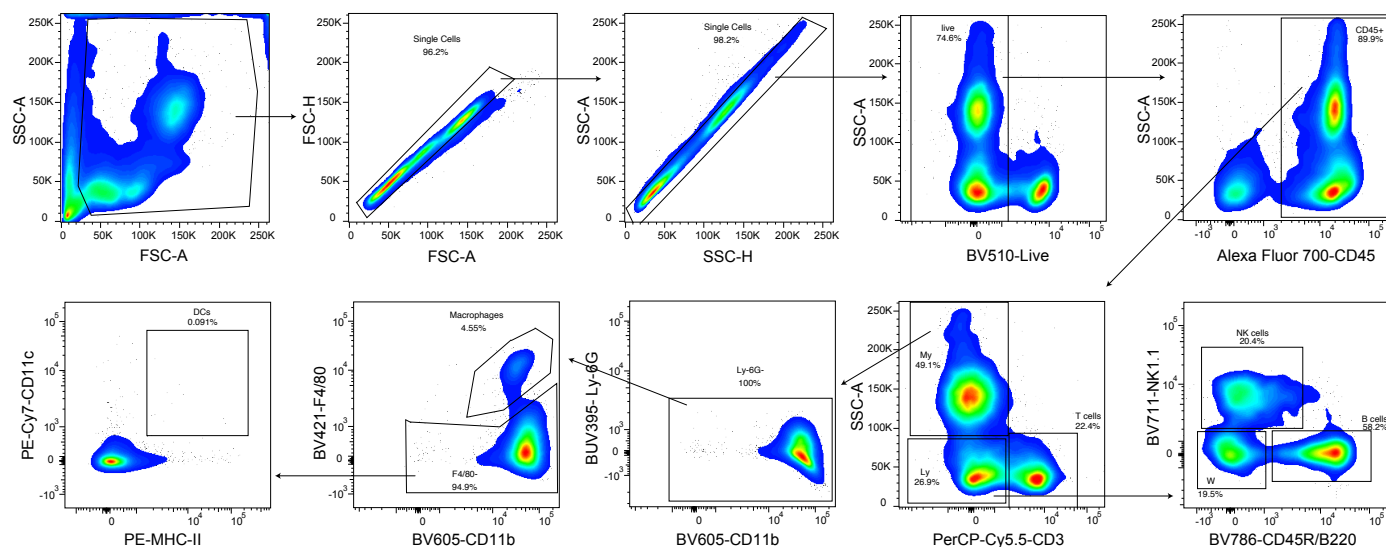

B

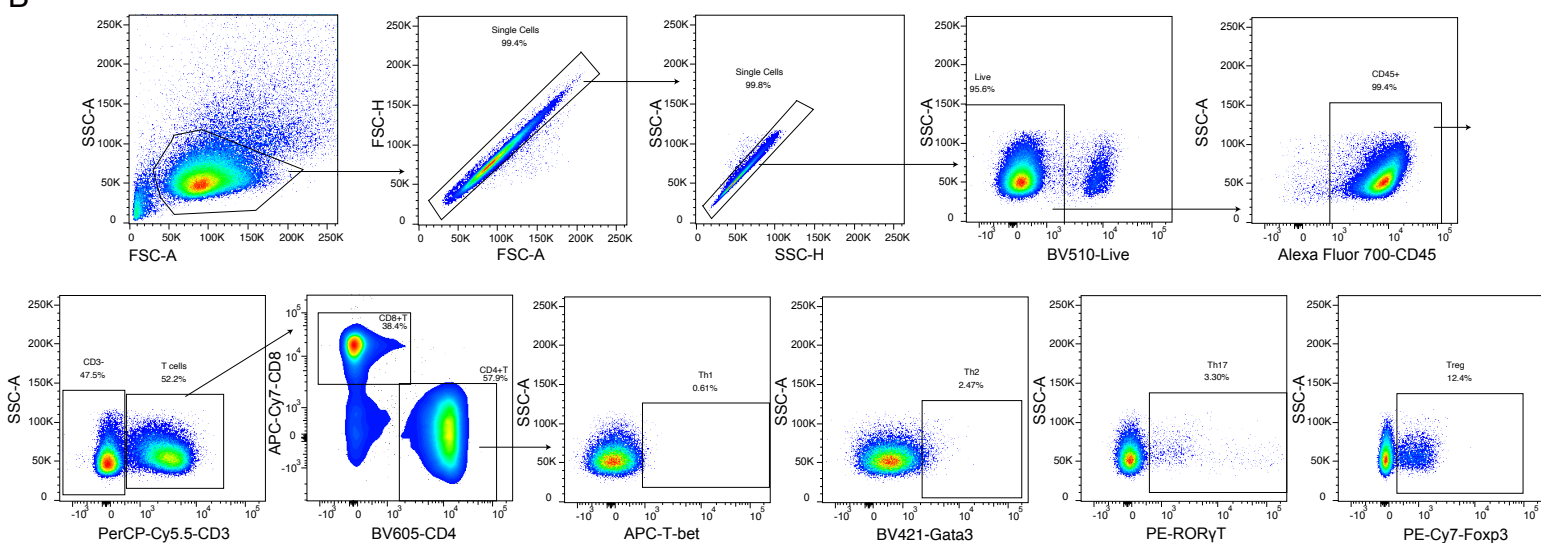

C

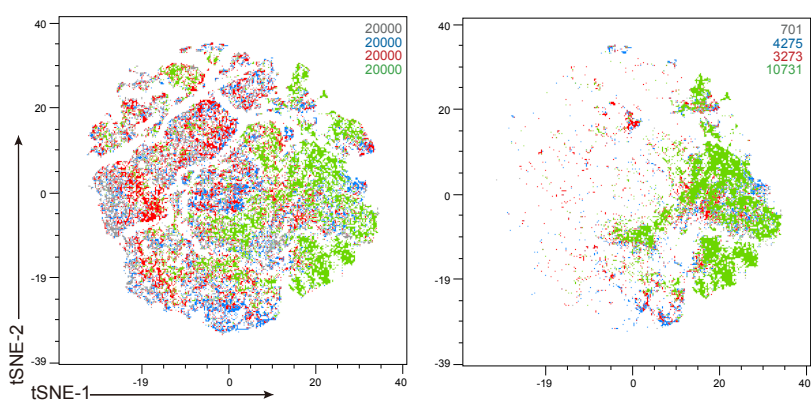

D

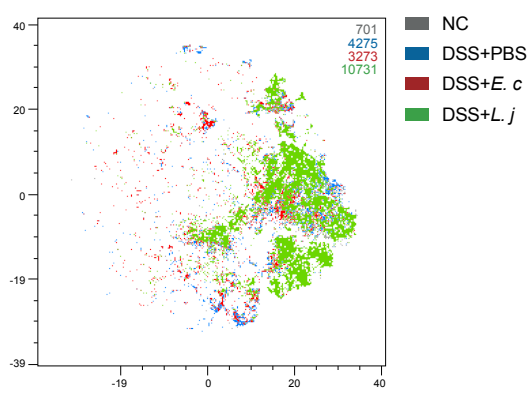

# Figure S3

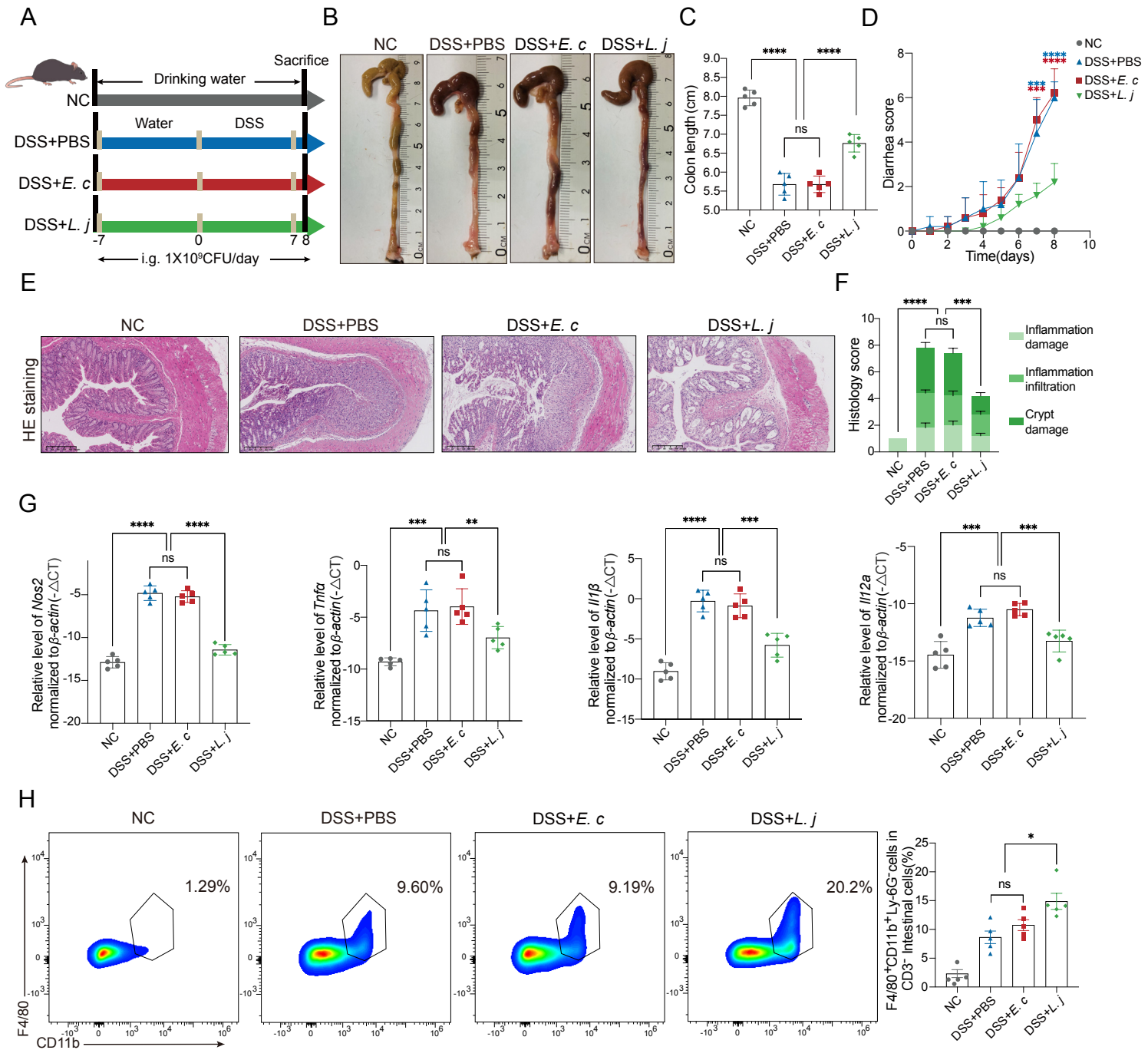

# Figure S4

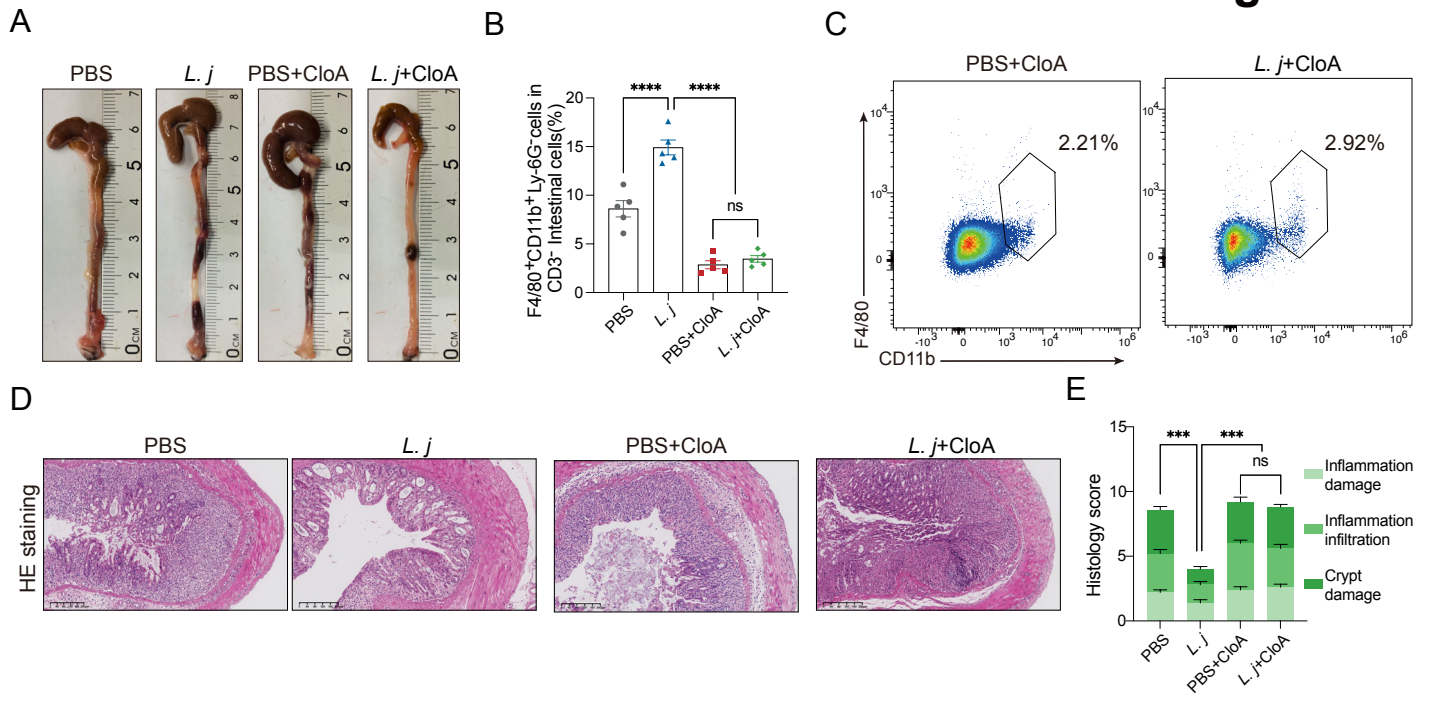

# Figure S5

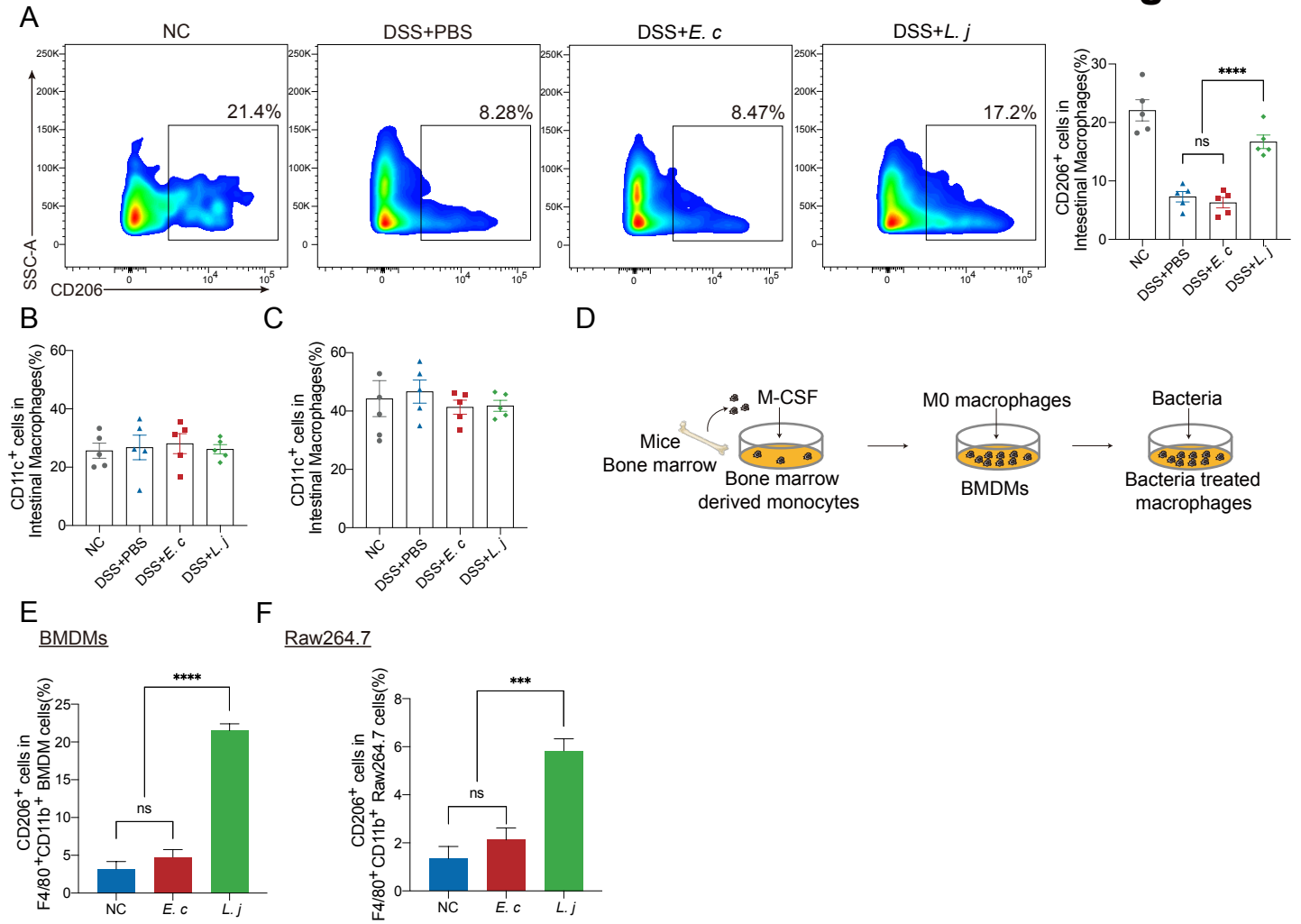

# Figure S6

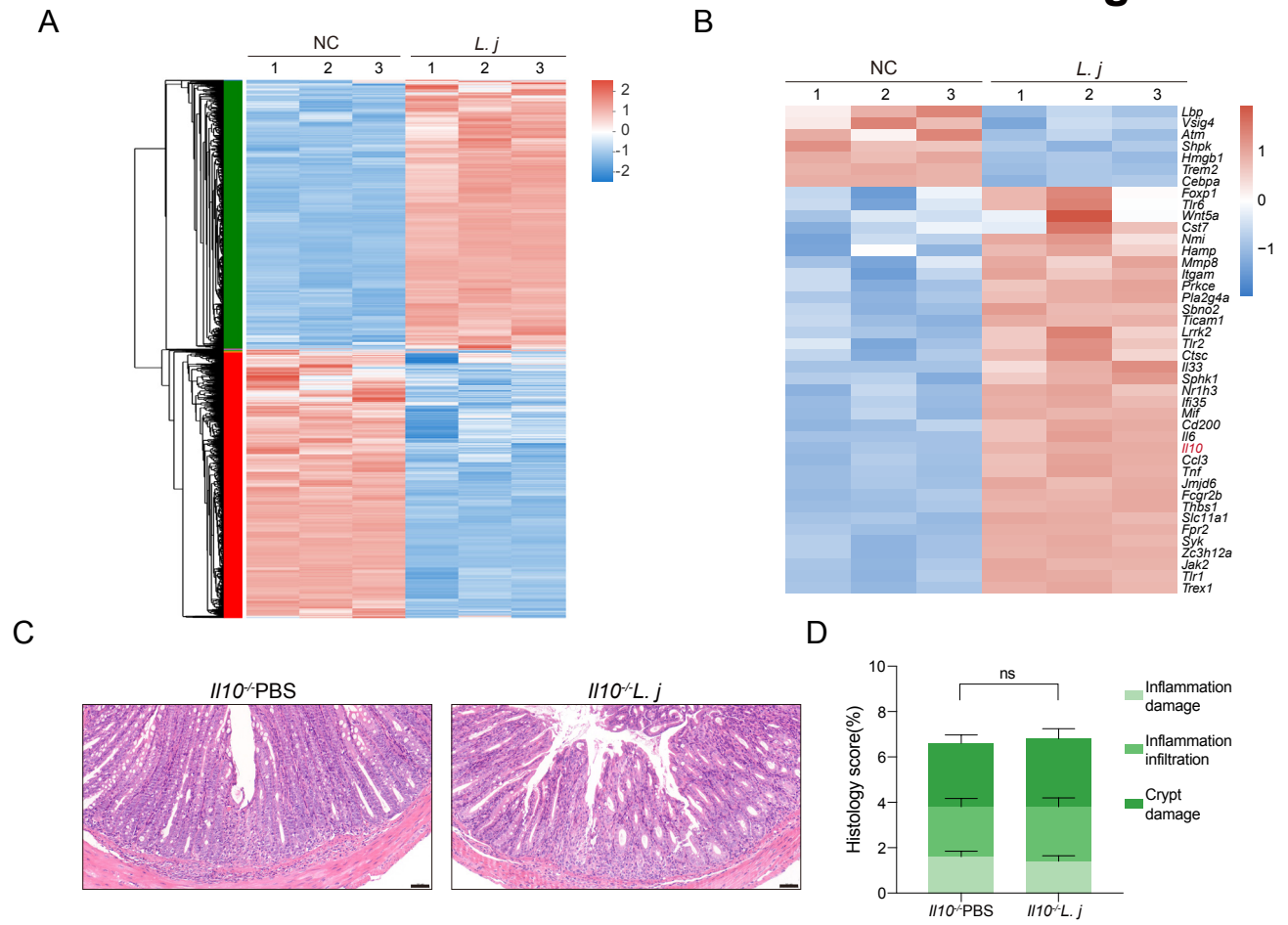

# Figure S7

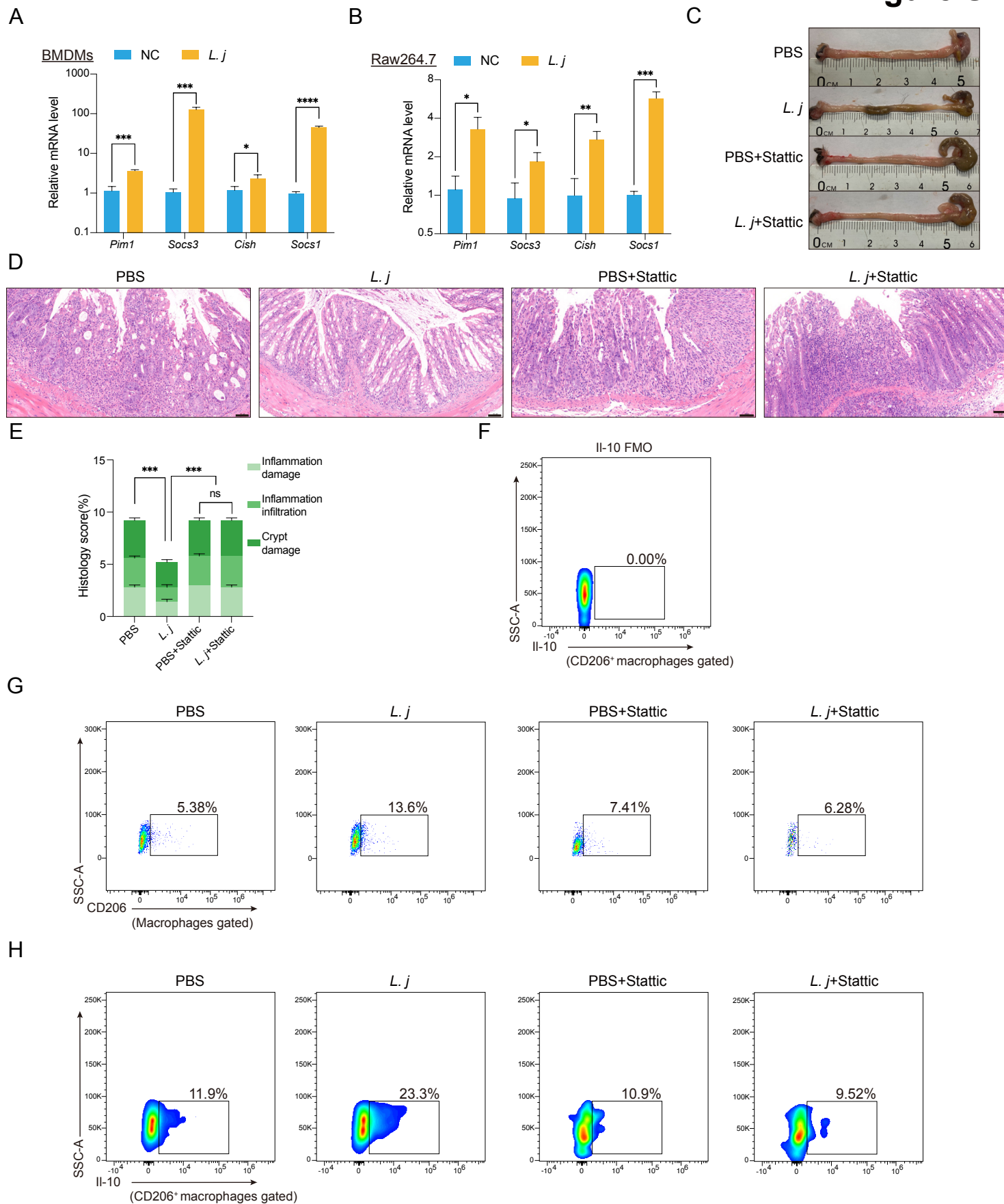

# Figure S8

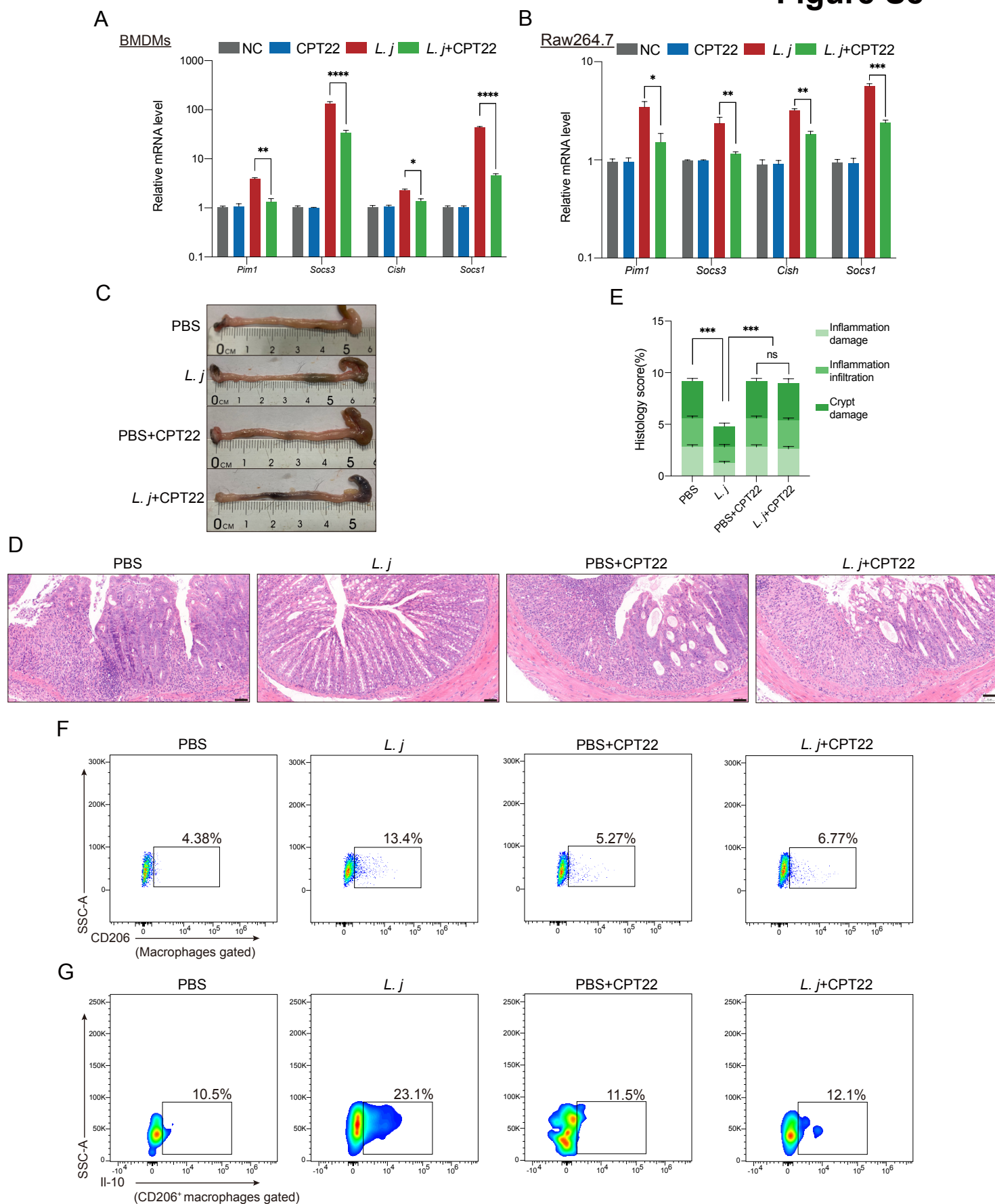

Supplement: Supplemental Material [file KGMI_A_2145843_SM4489.zip › Supplemental figure for KGMI20220604 revision.pdf]
